# A Rare Case of Gastric Trichobezoar Managed by Laparotomy

**DOI:** 10.7759/cureus.67357

**Published:** 2024-08-21

**Authors:** Aneesh Rao Nadipelli, Dileep Duggineni

**Affiliations:** 1 Department of General Medicine, Government Siddhartha Medical College, Vijayawada, IND; 2 Department of Internal Medicine, Government Siddhartha Medical College, Vijayawada, IND

**Keywords:** exploratory laparotomy, psychiatric comorbidity, trichotillomania, trichobezoar, gastric trichobezoar

## Abstract

Trichobezoars, rare gastrointestinal foreign bodies composed of ingested hair, typically affect females with psychiatric disorders such as trichotillomania and pica. This case report highlights the diagnostic and treatment challenges in an 18-year-old female presenting with a left upper quadrant mass and pain, who was found to have a large gastric trichobezoar. After diagnostic imaging confirmed the bezoar, it was surgically removed, and the patient had an uneventful recovery. Psychiatric follow-up was arranged to address the underlying disorders and prevent recurrence. This case underscores the importance of early recognition and management of trichobezoars to avoid serious complications.

## Introduction

Trichobezoar is a concretion of hair in the gastrointestinal tract that develops over a period of time. These masses can form due to the ingestion of hair, a behavior often seen in individuals with psychiatric disorders [1,2]. In the early stages, most cases of trichobezoar may not be identified because of their nonspecific presentation or may even not have any symptoms, making early diagnosis challenging [1]. Trichobezoars are almost always seen exclusively in females with underlying psychiatric illnesses such as trichotillomania, pica, obsessive-compulsive disorder, and a history of abuse [2-4]. Trichotillomania is a condition where individuals have an irresistible urge to pull out their hair, often leading to the ingestion of hair (trichophagia). Pica involves the consumption of nonfood items, including hair, which can contribute to bezoar formation. Obsessive-compulsive disorder may also lead to repetitive behaviors such as hair pulling and ingestion. Studies indicate that up to 90% of trichobezoar cases occur in young females with these underlying conditions [1-3].

When not recognized, trichobezoars continue to grow in size and weight, eventually causing gastrointestinal obstruction, which may extend into the small bowel [2,3]. This obstruction can lead to symptoms such as abdominal pain, nausea, vomiting, and even acute surgical emergencies. Although the prevalence of trichobezoar is low in humans, if not treated, the mortality rate is as high as 30% due to associated complications such as mucosal erosion, tissue destruction, perforation, and even death [5]. Complications such as gastric ulceration, peritonitis, and sepsis have been documented in severe cases where delayed diagnosis led to severe complications, emphasizing the need for awareness and early intervention in patients presenting with relevant psychiatric histories and gastrointestinal symptoms.

Diagnostic imaging, including ultrasound, CT scans, and endoscopy, plays a crucial role in identifying trichobezoars. Treatment typically involves surgical removal of the bezoar, followed by psychiatric evaluation and intervention to address the underlying psychiatric disorder and prevent recurrence. This highlights the importance of a multidisciplinary approach in the management of trichobezoar cases, involving surgeons, psychiatrists, and primary care providers to ensure comprehensive care and reduce the risk of recurrence. This case report discusses the diagnostic hurdles and the successful surgical approach for managing a rare gastric trichobezoar in an adolescent patient.

## Case presentation

Patient history

An 18-year-old female presented to the emergency department with a chief complaint of a left upper quadrant mass and pain persisting for two months. The patient reported experiencing early satiety and difficulty gaining weight, without any history of nausea, vomiting, fever, or changes in bowel movements. Additionally, the patient had a known history of trichophagia and pica (including ice and soil ingestion) but had not previously sought psychiatric consultation.

Physical examination

Physical examination was notable for pale conjunctiva and spots of baldness on the scalp. Abdominal examination revealed a mobile, noncompressible mass in the left upper quadrant with tenderness and no associated guarding.

Laboratory findings

Laboratory tests indicated mild anemia (hemoglobin: 8.7 g/dL) and leukocytosis (leukocyte count: 12,000 cells/μL). Serum electrolytes as well as liver and renal function tests were within the normal range (Table 1).

**Table 1:** Initial laboratory investigations obtained in the emergency department.

Test	Test result	Reference range
Hemoglobin	8.7 g/dL	12–16 g/dL
Leukocyte count	12,000 cells/μL	4,500–11,000 cells/μL
Platelet count	270 × 10^3^ cells/μL	150–450 × 10^3^ cells/μL
C-reactive protein	2.7 mg/L	<10.0 mg/L
Erythrocyte sedimentation rate	4 mm/hour	0 to 20 mm/hour
Blood urea nitrogen	13 mg/dL	7–20 mg/dL
Aspartate transaminase	13 U/L	5–34 U/L
Alanine transaminase	15 U/L	5–55 U/L
Serum creatinine	0.8 mg/dL	0.6–1.1 mg/dL
Serum sodium	138 mEq/L	136–145 mEq/L
Serum potassium	4.2 mEq/L	3.5–5.0 mEq/L
Serum chloride	98 mEq/L	95–105 mEq/L

Imaging results

X-ray erect abdomen demonstrated a normal bowel gas pattern. A contrast-enhanced CT scan of the abdomen revealed a large, free-floating solid mass distending the stomach and intraluminal space with mottled lucencies extending up to the proximal duodenum.

Management

A gastric bezoar was diagnosed and an upper gastrointestinal endoscopy was done which revealed a large gastric trichobezoar almost occupying the entire stomach. The duodenum was not visualized. In light of the large size of the bezoar and being adherent to the gastric wall, it was thought that endoscopic or laparoscopic removal would not be feasible without fragmentation and possible distal intestinal obstruction. Therefore, the patient was referred for surgical removal.

**Figure 1:**
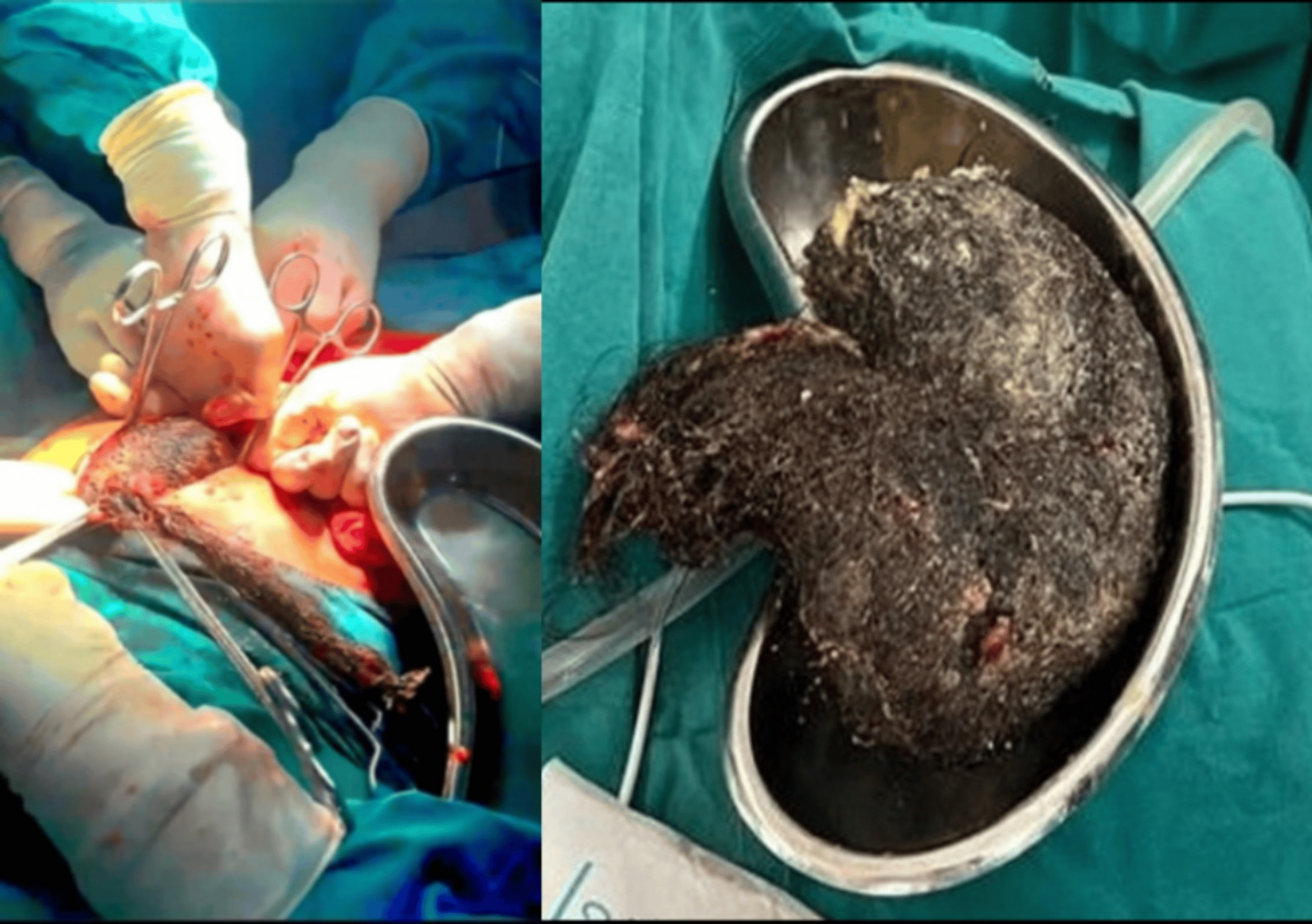
Intraoperative image showing the extraction of gastric trichobezoar through a gastrotomy (left) and the extracted specimen (right).

An exploratory laparotomy through an upper midline abdominal incision was performed. Following laparotomy, gastrotomy through the anterior wall of the stomach was performed. Intraoperatively, a large smoothly contoured mass was found occupying the bulk of the stomach extending into the first part of the duodenum, which was retrieved (Figure 1). The rest of the abdominal viscera were unremarkable. Double-layered closure of the anterior wall of the stomach was done with the placement of a single drain in the abdominal cavity followed by the closure of laparotomy. The postoperative course was uneventful. Oral feeding with iron supplementation was started on postoperative day five and the drain was removed on postoperative day seven. The patient was discharged after a psychiatry consultation for trichotillomania and pica, for which citalopram once a day was prescribed. The patient came for a follow-up one week after discharge with no new complaints.

## Discussion

A bezoar is an accumulation of foreign particles in the gastrointestinal tract over a period of time because of large-sized particles, gastric outlet obstruction, and intestinal stasis. Different types include phytobezoar (undigested vegetable fibers), trichobezoar (hair), and lactobezoar (milk/milk products). Many factors such as reduction in gastric acidity, gastrointestinal motility, and delayed gastric emptying have been implicated as contributory factors for bezoars [6]. In trichobezoars, the ingested hair follicles because of their smooth surface tend to avoid the peristaltic movements of the stomach and become embedded in the gastric rugae. The acid from the stomach acts on the hair follicles making them black and matted together with other follicles. Over time, food particles get stuck in the entangled hair follicles and cause halitosis (from bacterial colonization and fat fermentation) and bowel obstruction [7]. Factors associated with trichobezoar include pica, mental retardation, female gender, psychiatric illnesses such as obsessive-compulsive disorder, trichotillomania (plucking of hair leading to spots of baldness on the scalp), and subsequent trichophagia (ingestion of hair) [8,9].

A rare form of gastric trichobezoar is called Rapunzel syndrome, in which the tail of the bezoar extends distally into the small intestine, which may even extend until the ileocecal valve and may cause intestinal obstruction [7]. The signs and symptoms of gastric trichobezoar are often seen over a long period and are nonspecific, including epigastric pain, epigastric mass, nausea and vomiting, loss of appetite, early satiety, halitosis, weight loss, constipation, or diarrhea. A long-standing bezoar can also lead to complications such as gastric erosion and bleeding, obstructive jaundice, acute pancreatitis, and malabsorption-related complications such as iron deficiency anemia, megaloblastic anemia, and protein-losing enteropathy [10]. The gold standard for diagnosing trichobezoar is considered to be upper gastrointestinal endoscopy, but the most accurate test for determining the presence of trichobezoar is a CT scan of the abdomen, as it demonstrates mottled, nonhomogenous space-occupying lesions in the lumen with low attenuation and air trapping [11]. Because of the large dose of radiation through CT scans and most of the disease-containing population being young women of reproductive age, an MRI of the abdomen can be suggested [12,13].

Trichobezoars can be treated through endoscopic removal, laparoscopy, or laparotomy. Historically, endoscopic removal of trichobezoars has had limited success, with only small trichobezoars being effectively treated this way [14,15]. Repeated endoscopic attempts carry risks of iatrogenic complications such as esophageal perforation. Laparoscopy has been used successfully in some cases; however, it requires fragmenting the bezoar and removing it in chunks, which can be tedious and carries risks of gastric content spilling into the abdomen, leading to suboptimal outcomes, especially for large trichobezoars [16].

Laparotomy is considered the treatment of choice for trichobezoars due to its high success rate and relatively low complication rate [17]. A retrospective study conducted in 2005 confirmed that 34 cases of gastric trichobezoars were successfully treated with laparotomy followed by anterior gastrotomy [18]. In a case series by Mirza et al., which involved 17 patients (88% female), most presented with abdominal pain and vomiting. Severe complications, including small bowel obstruction and gastric perforation, developed in 41% of the cases. Rapunzel syndrome was identified in 54% of these patients. Fourteen patients underwent gastrotomy, while three required enterotomy, with one postoperative death and one recurrence reported [19]. In another case series by Haggui et al., involving six female patients presenting with epigastric pain, vomiting, and a palpable mass, trichobezoars were confined to the stomach in most cases. Surgical intervention was required for all patients, and despite failed endoscopic attempts in two cases, postoperative outcomes were favorable, with no recurrences [20].

The patient’s initially unclear history of trichophagia and the presence of a left upper quadrant mass with associated pain presented a diagnostic challenge, underscoring the importance of considering trichobezoars in relevant clinical presentations. Diagnostic imaging played a crucial role in identifying the large gastric trichobezoar, which led to prompt surgical intervention. The patient’s history of trichophagia and the presence of a left upper quadrant mass were classic indicators of a trichobezoar, emphasizing the importance of a thorough clinical evaluation.

## Conclusions

Trichobezoars, predominantly seen in women with psychiatric disorders, should be considered in the differential diagnosis of gastric outlet obstruction, especially in patients presenting with nonspecific gastrointestinal symptoms. Due to the often subtle and nonspecific nature of early symptoms, trichobezoars are typically diagnosed only when the condition has progressed to a severe stage, leading to significant morbidity. The most effective treatment currently available is surgical retrieval of the trichobezoar through laparoscopy followed by gastrotomy, fragmentation, and extraction for small trichobezoars, or laparotomy followed by anterior gastrotomy for large trichobezoars, which reliably removes the obstructive mass. However, to ensure long-term success and prevent recurrence, it is crucial to address and manage the underlying psychiatric condition, such as trichotillomania or pica. This requires a comprehensive, multidisciplinary approach involving both surgical intervention and psychiatric care. Therefore, early recognition, prompt surgical management, and ongoing psychiatric treatment are essential to effectively treat trichobezoars and improve patient outcomes.
